# Integrating whole-genome sequencing and epidemiology to characterise *Mycobacterium bovis* transmission in Ireland: a proof of concept

**DOI:** 10.1186/s13620-025-00321-3

**Published:** 2025-12-01

**Authors:** Nicola Harvey, Guy McGrath, James O’Shaughnessy, Jamie A. Tratalos, Brian Byrne, Adrian Allen, Robin Skuce, Kevin Kenny, Stephen V. Gordon, Damien Farrell

**Affiliations:** 1 Centre for Veterinary Epidemiology and Risk Analysis, School of Veterinary Medicine, University College Dublin, Belfield, Dublin 4, Ireland; 2 UCD School of Veterinary Medicine, University College Dublin, Dublin 4, Ireland; 3 DAFM Laboratories, Backweston Campus, Co. Kildare, Ireland; 4 Food Microbiology Division, Backweston Laboratory Campus, Department of Agriculture, Food and the Marine, Co. Kildare, Ireland; 5 Agri-Food and Biosciences Institute (AFBI), Veterinary Sciences Division, Belfast, UK; 6 UCD One Health Centre, University College Dublin, Belfield, Dublin 4, Ireland; 7 UCD School of Medicine, University College Dublin, Dublin 4, Ireland

**Keywords:** *Mycobacterium bovis*, Bovine Tuberculosis (bTB), Whole-genome sequencing (WGS), Epidemiological data, *Meles meles*, Cattle, Transmission, Ireland

## Abstract

**Background:**

In the Republic of Ireland, the herd-level incidence of bovine tuberculosis (bTB), caused by *Mycobacterium bovis*, reached 6.40% by June 29th 2025, highlighting bTB’s risk to animal health, biosecurity and the economy. The complexity of bTB epidemiology, driven by multiple host species, undetected transmission and incomplete diagnostic sensitivity, makes surveillance and control challenging. Pathogen whole-genome sequencing (WGS) can clarify transmission dynamics but is constrained by the slow, variable mutation rate of *M. bovis*.

This pilot case study integrates WGS with epidemiological data to elucidate transmission event pathways and could be a starting point for future automation. A decision-tree framework was developed to classify likely transmission event pathways by integrating *M. bovis* WGS sourced from the BTBGenie research project and epidemiological data. As proof-of-concept, one farm with multiple isolates was randomly selected from a national WGS database. Twenty-eight near-identical isolates (pairwise ≤ 3 SNP divergence), from other herds, were identified across databases of the Republic of Ireland and Northern Ireland, with full metadata was available for 16 isolates. These were analysed using TracebTB, a research tool linking national animal health, movement, wildlife and land management databases.

**Results:**

Transmission event pathways for this case herd were classified as “local area transmission” (43.75%), “within-herd transmission” (12.5%), and “cattle movement-associated transmission” (also 43.75%), divided into between-herd (25%) and within-herd (18.8%) transmission. No evidence of residual within-herd transmission was found in this herd. Homebred animals served as spatial anchors, delineating the cluster’s ‘home range’ or kernel. A spatially distant homebred case, alongside the overall home range size, suggested an undetected movement-associated transmission event, likely via undetected carriers.

**Conclusions:**

Integrating WGS with detailed epidemiology enables identification of probable bTB transmission event pathways, revealing undetected infections and highlighting biosecurity concerns associated with undetected carriers. The decision-tree framework provides a scalable approach for retrospective outbreak investigation, targeted surveillance, and efficient resource allocation, particularly in high-risk systems such as Controlled Finishing Units.

These findings highlight the importance of transboundary collaboration in persistent bTB hotspots. Automating this approach could support validation of disease epidemiological models, guide targeted interventions, and optimising resource allocation, supporting Ireland’s goal of bTB eradication.

**Supplementary Information:**

The online version contains supplementary material available at 10.1186/s13620-025-00321-3.

## Background

Bovine tuberculosis (bTB), caused mainly by *Mycobacterium bovis*, remains a significant challenge to both human and animal health. *Mycobacterium bovis* is a slow growing member of the *Mycobacterium tuberculosis* complex (MTBC), characterised by chronic diseases that tend to include a latent period [1]. In the Republic of Ireland, the herd-level incidence of bovine tuberculosis (bTB), caused by *M. bovis*, increased from a historic low of 3.27% in 2016 to 6.40% by June 29th 2025 [2], highlighting bTB’s risk to animal health, biosecurity and the economy [3]. Routine surveillance for bTB in Ireland includes an annual full herd test for all cattle, using the Single Intradermal Comparative Tuberculin Test (SICTT), the statutory diagnostic method mandated under Animal Health Law Regulation 2016/429. However, in Ireland up to one third of bTB outbreaks are first identified during abattoir surveillance, when lesions consistent with bTB are confirmed at post mortem examination, despite animals testing negative or sometimes inconclusive by SICTT [4]. Despite global eradication efforts, bTB persists in many regions, largely due to the complex epidemiology of *M. bovis* and gaps in understanding its transmission dynamics [5, 6]. In particular, the sensitivity of current diagnostic tests is limited, particularly in early infection stages [7, 8] leading to challenges in disease surveillance and control.

The epidemiology of bTB is further complicated by the diverse range of hosts that can be infected with *M. bovis*. These hosts includes cattle and wildlife, such as badgers (*Meles meles*) and deer in the Republic of Ireland [9]. Timing of infection does not therefore always align with bacterial isolation, making it difficult to trace the introduction of new infections [10]. The ability to accurately trace infection sources is crucial for effective bTB control, as it enables the identification of key transmission event pathways, facilitates targeted intervention by identifying critical control points, and enhances the ability to forecast disease spread and evaluate intervention efficacy [11, 12].

### Whole genome sequencing and its role in bTB Epidemiology

Advances in molecular epidemiology, particularly the application of pathogen whole-genome sequencing (WGS), have greatly enhanced the study of infectious disease transmission as WGS enables high-resolution discrimination of transmission pathways [13]. The most discriminatory WGS analysis method is by the alignment of reads to an annotated *M. bovis* AF122/97 reference genome consisting of over 4.3 × 10^6^ bases [14]. The variation in the genomic sequence of each isolate can be measured by detecting all Single Nucleotide Polymorphisms (SNPs) across the genome, having masked well-documented regions of the genome which contain highly repetitive sequences [15]. An alternative approach uses the concept of core genome Multi-locus Sequence Typing (cgMLST), counting allelic differences instead of SNPs. This can be convenient for typing but has somewhat lower resolution than counting every SNP in the genome [16]. Both these approaches to measuring genetic variation serve as molecular markers that can be used to reconstruct transmission chains with greater precision than traditional genotyping methods [17]. Thus, WGS allows researchers to differentiate bacterial isolates and more reliably infer evolutionary relationships between them [13].

An initial research study used WGS to characterise *M. bovis* isolates from cattle, deer and badgers in an area of Co. Wicklow [11]. The BTBGenIE research project was then funded by the Department of Agriculture, Food and the Marine (DAFM) to study the genomic epidemiology of *M. bovis*, including a focus in Co. Monaghan, using isolates from cattle and badgers. DAFM now routinely performs WGS of *M. bovis* isolates obtained from the national cattle herd in the Republic of Ireland as well from wildlife. Analysis of this national dataset has revealed a high level of genetic diversity in circulating *M. bovis* strains, with observed SNP distances of up to 490 (median 252) between some isolates [15]. The nucleotide substitution rate of *M. bovis* is estimated to be 0.5–1 SNPs per year [5, 12] which is substantially lower than many other bacterial pathogens [18]. Furthermore, fixation rates are known to fluctuate over short timescales due to bacterial latency and other factors [19]. As a result, some genetically linked isolates can exhibit identical SNP profiles over several years, whereas others may accumulate multiple SNPs within a shorter timeframe [12, 20]. This variability complicates the determination of what constitutes a recent transmission event. However, despite these caveats, the general rule of thumb remains—the smaller the number of SNPs observed between isolates, the more likely it is they could be related by an infection transmission event.

### Defining transmission cut-offs in WGS analysis

Defining an appropriate SNP threshold for recent transmission is a fundamental challenge in the use of WGS for epidemiological investigations. Studies indicate that within-herd SNP diversity in *M. bovis* is low, with 40% of within-herd isolates exhibiting no divergence and 86% accumulating three or fewer SNPs relative to any other isolate found within the same herd in the same breakdown [21]. Research on *M. tuberculosis,* closely related to *M. bovis*, has shown that using a threshold of < 5 SNPs is consistent with transmission events occurring up to ten years in the past [16, 19]. However, given that the average life expectancy of Irish cattle is between 39 and 42 months [22, 23], a broader SNP threshold might overestimate the likelihood of direct transmission. Additionally, sequencing error rates and homogeneity in outbreak-level WGS data necessitate careful consideration when interpreting genomic similarities between isolates. Based on these factors, a cut-off of ≤ 3 SNPs has been adopted in this case-study as a conservative estimate for defining recent transmission in Irish cattle herds.

Despite the high resolution of WGS, the slow and variable evolutionary rate of *M. bovis* limits its ability to resolve fine-scale transmission events [24]. To address these limitations, studies have integrated genomic data with epidemiological metadata such as cattle location, movement, and test records to identify longer distance transmission events in epidemic bTB outbreaks [25]. The combination of WGS data with life events, such as animal movements, lifespan and diagnostic testing histories, can provide stronger evidence for transmission links or help rule out potential connections that appear plausible based on epidemiological metadata alone, and vice versa [1, 26–29].

### Objectives of this pilot study

This pilot study relies on WGS data generated by the BTBGenIE research project to investigate a small number of bTB outbreak herds with well recorded classical epidemiological metadata (cattle movement records, lifespan data, testing history and the geographical distribution of sampled wildlife). The study aims to develop a decision tree framework that integrates these epidemiological metadata with current Irish *M. bovis* WGS data. We sought to evaluate how the latter might enhance disease outbreak investigations, more robustly informing on probable transmission routes. Specifically, this integrative approach was used to characterise five key *M. bovis* transmission event pathways [1, 24];

1. Within-herd transmission event – Transmission event between animals within the same herd or simultaneous exposure to an infectious animal.
2. Residual within-herd transmission event – Persistence of *M. bovis* within a herd over more than one testing cycle, potentially due to undetected transmission events between infected animals or due to environmental reservoirs.
3. Local transmission event – Transmission event involving neighbouring herds, either directly through cattle movement or contact and/or indirectly via wildlife reservoirs such as badgers.
4. Transmission event associated with cattle movement between herds – Transmission event linked to the trade and transport of cattle between herds.
5. Transmission event associated with cattle movement within herds**—**Transmission event associated with movement between discrete land fragments associated with one herd.

As a proof of concept, this study applied the decision tree approach to a case study involving a small group of animals infected with nearly genetically identical (pairwise distance of ≤ 3 SNPs) *M. bovis* isolates. By integrating WGS data with classical epidemiological information, we aimed to reconstruct transmission event pathways with greater certainty than applying classical epidemiology alone allows, thereby contributing to the refinement of bTB control strategies in Ireland.

By enhancing our ability to differentiate between transmission event scenarios and thus determine their probable relative contributions, this study aimed to provide a robust framework for forensic-level epidemiological analysis of bTB outbreaks. The insights gained will provide data-driven farm-specific interventions, and support policy development aimed at reducing bTB prevalence and refining risk-based surveillance. They may also inform key variables for epidemiology models and help to evaluate output from such models. Together, these contributions may improve the overall effectiveness of the bTB eradication program.

## Methods

Cattle that are either Single Intradermal Comparative Tuberculin Test (SICTT) positive, or SICTT negative but disclosed a tuberculous lesion(s) at inspection post-mortem are defined as reactors*.* The BTBGenIE study used samples collected at slaughter for cattle sourced from Co. Monaghan, Sampling occurred between 2019 and 2021. Badgers culled in Co. Monaghan in 2018 and 2019 were submitted to either Sligo Regional Veterinary Laboratory or the Irish Equine Centre for necropsy and tissues were collected for culture. *M. bovis* isolates from cattle and badgers were heat killed and sequenced at University College Dublin or in DAFM laboratories [15].

For interactive investigation of clusters, we used TracebTB [30], a prototype interactive Python web application developed in-house. This tool is designed to facilitate microbial forensic epidemiological investigation of *M. bovis* transmission events by integrating WGS data with metadata encompassing national cattle movement, bTB diagnostic testing data, land parcel data and wildlife capture datasets. The tool displays any selected samples as locations on an interactive map. Other elements, such as land parcels, cattle movements and cattle lifespans, can be overlayed on the map. Genetic relatedness is displayed dynamically as a phylogenetic tree or minimum spanning representation. Sample points may be coloured by any metadata field. Sample selection can be done in several ways: through a text search, selection from a list of samples or by group selection (e.g. all samples from a given SNP cluster or herd). In this way we intend to build an application suitable for local and regional analyses.

### Data sources

TracebTB integrates information from multiple databases maintained by DAFM, including:

- Animal Health Computer System (AHCS): Contains individual animal identification records, diagnostic bTB testing history (Single Intradermal Comparative Tuberculin Test (SICTT) and Gamma Interferon (GIF) assay results), and post mortem findings.
- Animal Identification and Movement system (AIM): Contains the date of birth of each bovine animal in the national herd. It also contains all registered cattle movements including farm-to-farm transfers, between herd movements following cattle market (mart) sales, and end of life movements to slaughterhouses or knackeries.
- Land Parcel Identification System (LPIS): Provides spatial data linking specific land parcels associated with uniquely identified herds.
- Wildlife Administration Unit Software (WAU): Contains records of badger capture and sampling locations, contributing to investigations of wildlife-mediated transmission.
- *M. bovis* WGS dataset: Contains WGS sequences of *M. bovis* isolates obtained from cattle and badgers from the Republic of Ireland and Northern Ireland.

A number of *M. bovis* samples collected between 2017 and 2023 from the Republic of Ireland underwent WGS at the Central Veterinary Research Laboratory, DAFM Laboratories. This dataset was created during the BTBGenIE research project, (Department of Agriculture, Food and the Marine’s Competitive Research Funding Programme grant 20,194,404). The area sampled was selected due to bTB prevalence and the presence of a border with Northern Ireland. Within this context we selected a cluster for this study.

### SNP-based clustering

A SNP alignment was calculated from the alignment of all samples to the *M. bovis* AF2122/97 reference genome and subsequent variant calling as detailed by O’Shaughnessy et al. [15]. A maximum likelihood all-Ireland phylogeny was built from the SNP alignment. In our typing system, SNP differences are identified from the phylogeny using the Fastbaps [31] package in R. However, when considering recent transmission events these clades are too large. We therefore defined clusters of interest in an *ad-hoc* manner in TracebTB by choosing samples in the initial herd of interest and then identifying all samples ≤ 3 SNP, using the pairwise SNP distance matrix derived from the alignment.

### Decision tree approach to transmission classification

**Fig. 1:**
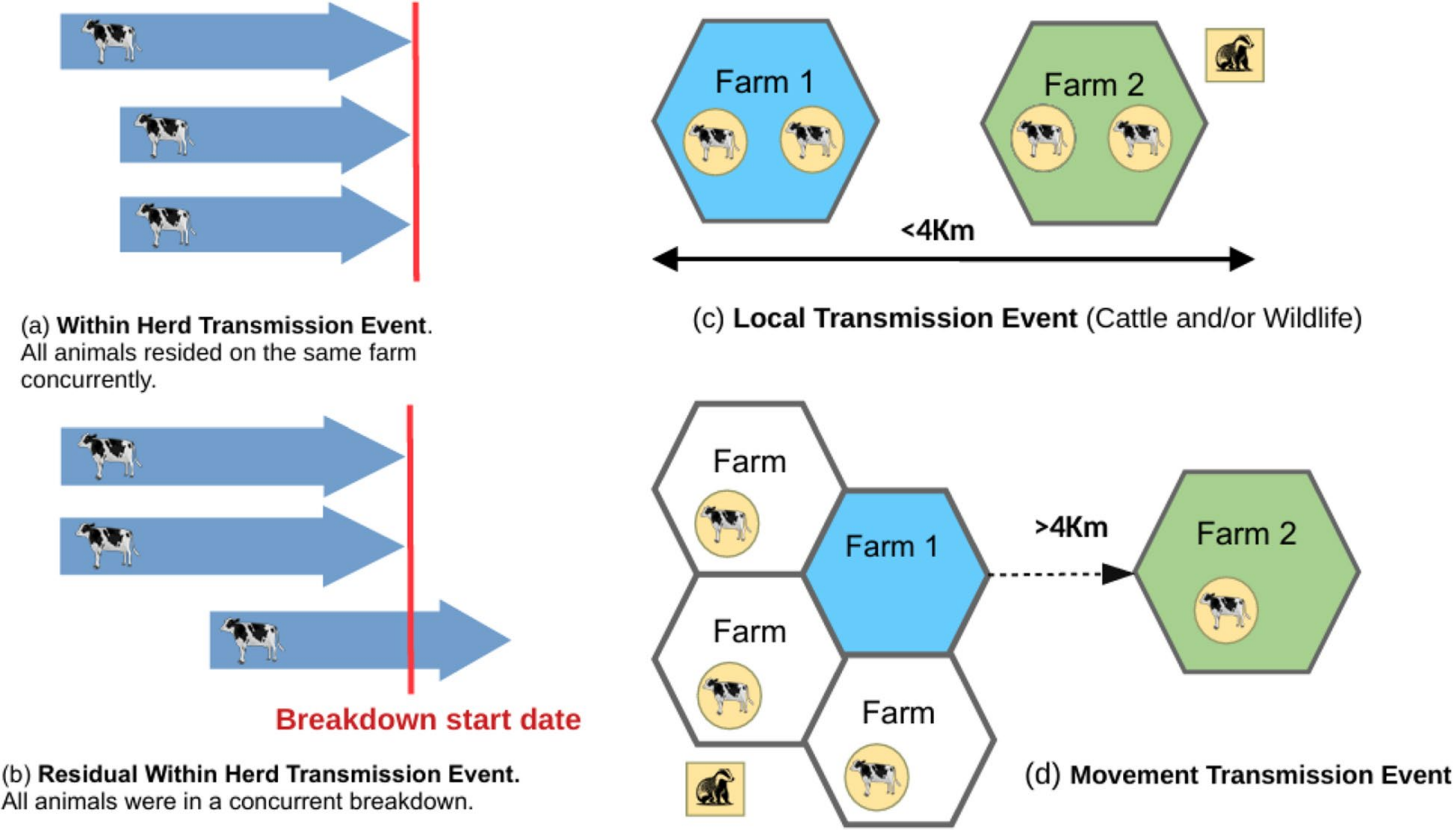
Transmission event pathways integrating WGS data and epidemiology data, where the “breakdown start date” refers to the test date on which the animal was disclosed as bTB positive (either ante or post mortem). Different colours represent the land parcels associated with the farms where WGS isolates were obtained. The badger icon represents WGS isolates from badgers, while the cow icon represents WGS isolates from cattle. The colour background on both icons denotes genetically near-identical M. bovis isolates (≤3 SNPs)

As a proof-of-concept, one farm with multiple contemporary *M. bovis* isolates was randomly selected from a national WGS database. Using the isolates identified on that farm as the index cluster, 28 nearly identical isolates (pairwise ≤ 3 SNP divergence) were identified across WGS databases from both the Republic of Ireland and Northern Ireland. The small sample size introduces sampling bias and limits the generalisability of the findings. Furthermore, comprehensive metadata (covering lifetime history, movement and residency) were only available for 16 of the 28 isolates. Despite these limitations a decision tree (Supplementary Figure A) was developed to systemically evaluate potential *M. bovis* transmission event pathways using TracebTB. For the purpose of this study, a homebred animal was defined as one with only a single recorded movement in its lifetime (i.e. to slaughter). The decision-tree framework considered five primary transmission event pathways (Fig. 1), classifying cases based on genetic similarity (≤ 3SNP), spatial proximity, temporal patterns and recorded movement history.

### Classification of transmission pathways

#### Within-herd transmission event

Cases were classified as “within-herd transmission event” when cattle within a single herd at the same time had genetically near-identical *M. bovis* isolates (≤ 3 SNPs). This scenario suggests that the infection of the cattle originated from a common infectious source, either cattle or wildlife, or that a direct or indirect transmission event occurred between cattle in the herd (Fig. 1a). This classification is critical for understanding the persistence of *M. bovis* within closed herds and evaluating within-farm risk factors.

#### Residual within herd transmission events

Cases were classified as “residual within-herd transmission events” when genetically identical isolates (≤ 3 SNPs) re-emerged in a herd < 42 months after it had regained Officially TB-Free (OTF) status, i.e. all herd tested negative for bTB in two SICTT, 60 days apart. This scenario indicates undetected persistence of *M. bovis*, potentially due to untested carriers, reactivation of a previous infection, or environmental contamination (Fig. 1b). To differentiate between true residual infection and new introductions, genetic divergence was considered. Given the estimated nucleotide substitution rate of *M. bovis* (0.5–1 SNP per year) [1, 12] and the average lifespan of Irish cattle (42 months) [32], a threshold of > 3 SNPs between isolates was used to exclude residual within-herd transmission. A higher SNP difference suggests an external introduction rather than persistence of a previous variant within the herd.

#### Local area transmission event

Transmission was classified as “local area transmission event” when genetically near-identical (≤ 3 SNPs) isolates were detected in animals from herds located within a 4 km radius (Fig. 1c). This criterion was based on epidemiological evidence indicating that badgers, a known wildlife reservoir for *M. bovis*, have an average ranging distance of < 2 km [33]. Furthermore, 4 km gave the best explanatory power in models measuring the relationship between bTB breakdown and bTB in neighbouring herds [23]. The 4 km threshold was chosen to account for both direct transmission between neighbouring cattle herds and potential badger-mediated spread.

#### Transmission event associated with cattle movement (> 4 km) between herds

In Ireland, herd owners are legally required to report cattle movements to AIM. Movements may occur when animals are sold at a mart, privately sold between two farmers, or move temporarily to another farm. This classification was assigned when WGS data showed genetically identical isolates (≤ 3 SNPs) were recovered from cattle that had resided in, or nearby herds, that this animal had previously resided in, before their infection was detected in their final herd (Fig. 1 d). In such cases, the transmission event likely occurred before the animal left the original herd, but detection only occurred after movement. This pathway is particularly relevant for understanding how bTB spreads through trade and temporary rearing arrangements.

#### Transmission event associated with movement within herds

Many farms in Ireland operate on non-contiguous land parcels [22, 34], where cattle are moved between the home farm (main holding) and outside fragments (additional land used for grazing). Unlike inter-herd movements, movements between land parcels are not required currently to be reported to AIM, making them difficult to track through conventional movement data.

Transmission events associated with movement within a herd was classified under this category, if genetically identical isolates (≤ 3 SNPs) were also found in other cattle from locations near outside land fragments, but not near the home farm. Such cases suggest undocumented (not officially recorded) within-herd movement played a role in the transmission event. This classification underscores the need to consider land-use patterns and potential gaps in movement recording when interpreting transmission dynamics.

This herd-level approach represents a significant advancement in the use of WGS for bTB epidemiology; it provides a consistent data-driven framework for attributing relative contribution of different infection sources of bTB.

## Results

### Datasets

Between 2017 and 2023, the AHCS database recorded 147,391 bTB-positive reactors. Of these 46,390 animals had bTB lesions confirmed following slaughter. In addition, 14,205 cattle that tested negative or inconclusive at their most recent SICTT were found to have bTB-positive lesions detected at routine *post mortem* examination upon slaughter. The current *M. bovis* WGS dataset contains sequences sourced from the BTBGenIE research project, which included tissue sampling from 1,395 cattle and 369 badgers, resulting in 271 bovine and 82 badger *M. bovis* isolates. The WGS dataset also contains sequencing data for *M. bovis* isolates collected from cattle and wildlife across the Republic of Ireland. As of 2025, this dataset contains 4,809 sequenced isolates:

o 4,255 bovine-derived isolates from 2,909 unique herds
o 554 badger-derived isolates
o An additional 642 sequences were provided by Agri-Food and Biosciences (AFBI) in Northern Ireland.

### Identification of study group

**Fig. 2:**
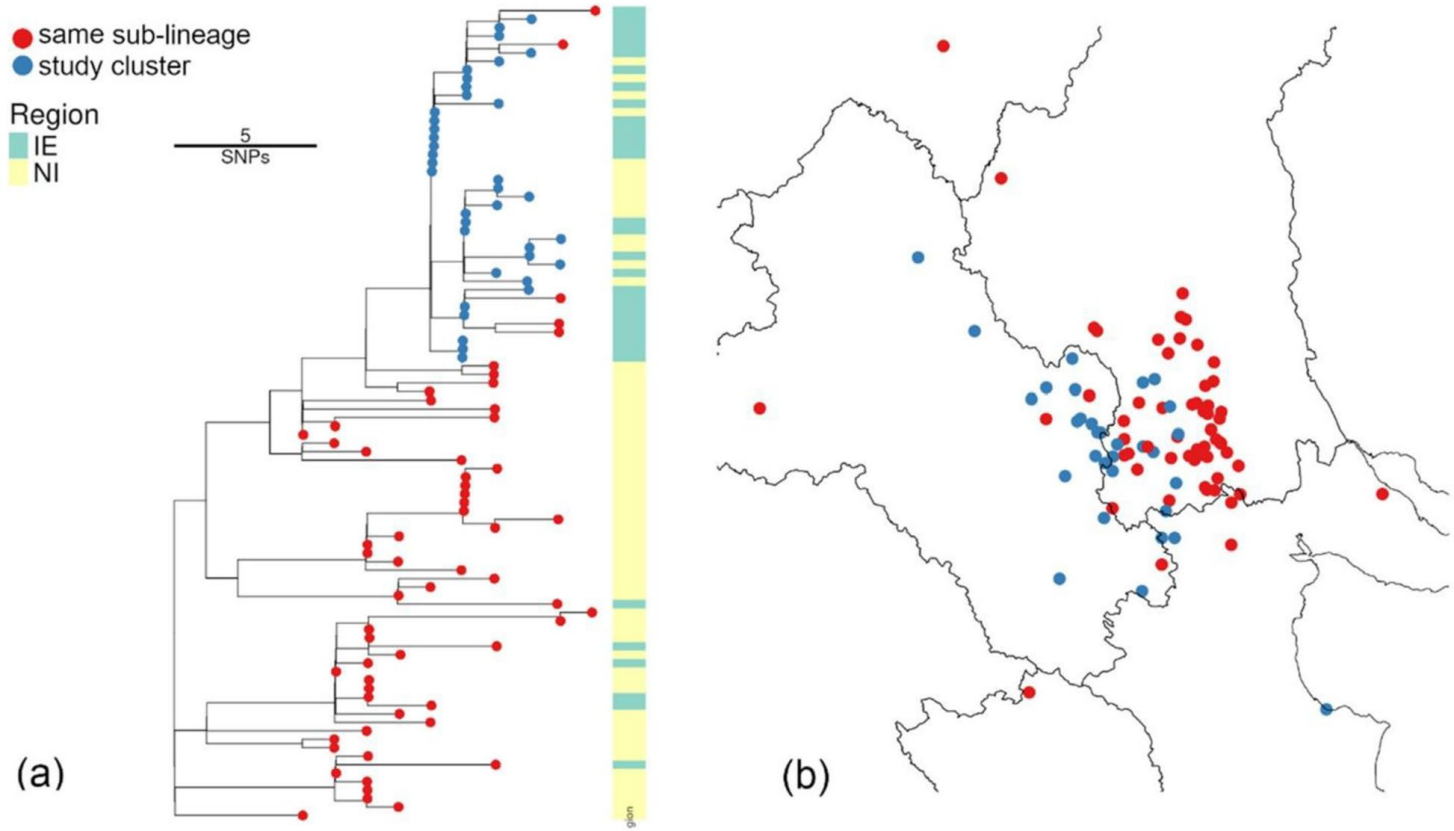
Genetic context for the study group shown in the sub-lineage phylogeny in (**a**). The isolates from this study are shown in blue in both plots. (**b**) shows the locations for these samples, clearly indicating the geographic localisation of the sub clusters, though with significant overlap. Such spatial patterns are common within lineages in Ireland. In order to facilitate detailed mapping, distant outliers, including one isolate submitted from a farm in Co. Donegal, are not shown

This pilot study focused on the WGS data obtained from two cattle in the same index herd. The herd was randomly selected from herds in Co. Monaghan, which had WGS data available from more than one animal. The animals were submitted for slaughter simultaneously in Spring 2019 and were part of the BTBGenIE study. Both animals exhibited near-identical WGS profiles (≤ 3 SNPs). An interactive query of the WGS database identified 28 additional isolates with near-identical sequences (≤ 3 SNPs, range 0–7 SNPs). These samples are contained inside a larger sub-lineage identified from the all-Ireland phylogeny [15]. This sub-lineage is shown in Fig. 2 with the study cluster indicated in blue. The majority of samples are located in Armagh. In order to facilitate detailed mapping, outliers, including one isolate submitted from a farm in Co. Donegal, are not shown. Note that there are several tips in the study group strain not included in the study (the red tips) as they did not satisfy the selection criteria (≤ 3 SNPs of index herd samples).

**Fig. 3:**
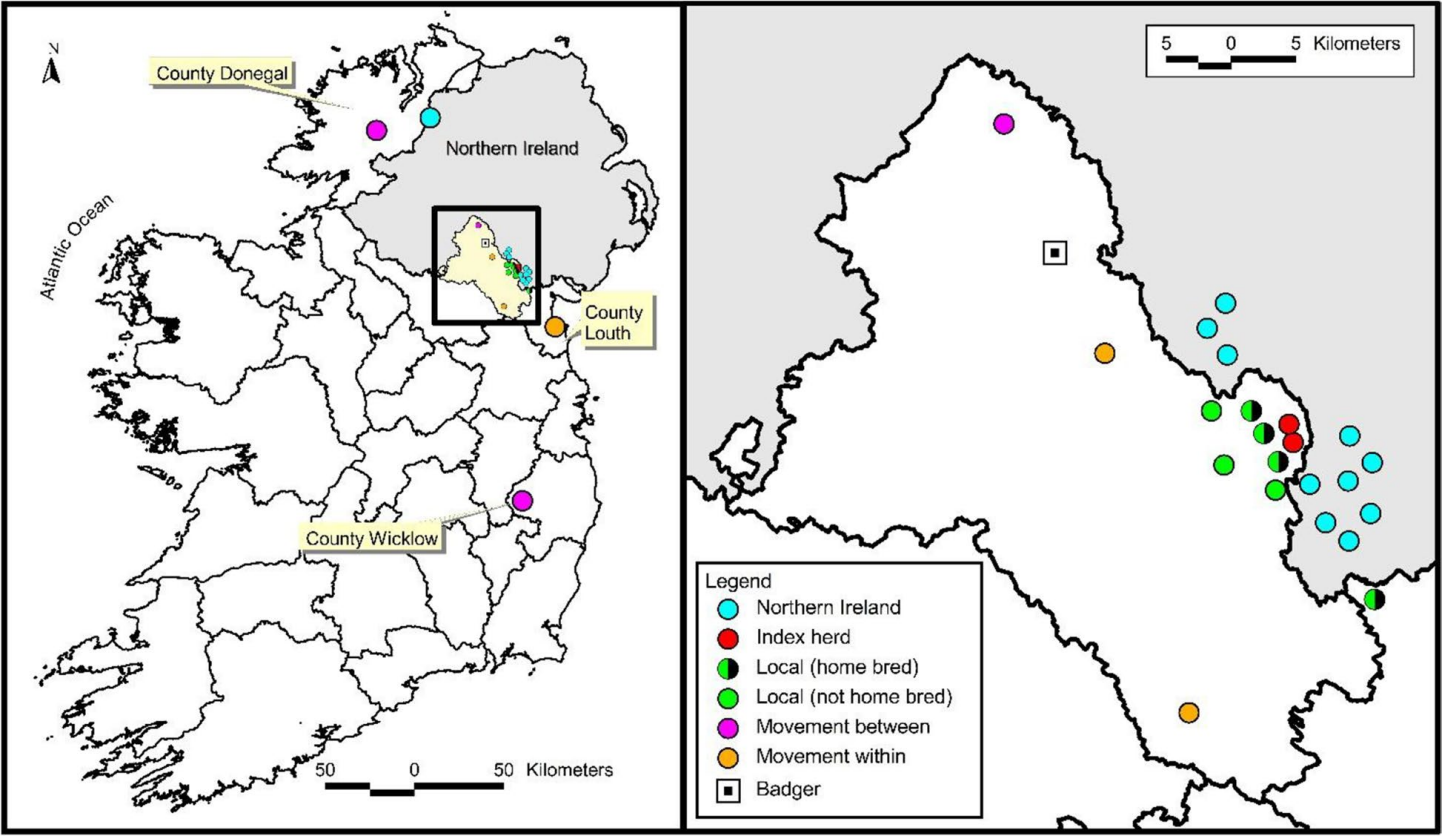
Approximate location of 28 genetically identical isolates with WGS data, where turquoise = N.I. samples, red= index herd, green & black = homebred local transmission event, green= local transmission event, pink= transmission event associated with movement between herds and orange = transmission event associated with movement within farm. Black & white square = badger

Of the study group, one isolate was excluded due to missing location data. A further 11 isolates located in Northern Ireland were only subjected to spatial analysis as final location data were the only metadata available for those isolates. The remaining 16 (28 minus 12) isolates were subjected to further epidemiological analysis (Fig. 3). Apart from the index herd which had two isolates, there was only one isolate per herd. Of these, 15 originated from cattle and one from a badger. Twelve of the cattle isolates and the badger isolate were related to the BTBGenIE study and originated from animals in herds in Co. Monaghan; the single badger sample was also from Co. Monaghan. Five animals classified as homebred were from farms located in Co. Monaghan (*n* = 3), Co. Donegal (*n* = 1), and Co. Louth (*n* = 1). Firstly, an animal was classified as homebred if it remained in the submitting herd from birth to slaughter, without any recorded movement to another herd. Transmission event classification was based on whether genomic and epidemiological evidence supported one of five possible transmission event pathways (Fig. 1). The decision tree framework developed for this study (Supplementary Figure A) was applied to classify transmission event pathways.

### Within-herd transmission event

Two isolates (Red points in Fig. 3 Plot B) were obtained from animals X and Y which were submitted contemporaneously for slaughter in Spring 2019, from the herd under investigation. Both animals tested bTB lesion-positive at post mortem inspection, and WGS data was obtained from those lesions.

- Epidemiological records show that both had resided on three separate farms in their lifetimes.
- Animal X was born 05/03/2017 and Animal Y was born on 08/03/2017.
- The herds they were born in were distant (> 50 km) from one another.
- Both animals each moved over 50 km to the same farm in Spring 2017, just one week apart (25/03/2017- Animal Y vs 01/04/2017-Animal X).
- In Autumn 2018, they both relocated together to the submitting herd, < 1 km away.

Since the two isolates have near-identical genomic profiles (≤ 3SNP), the transmission event was classified as a “within herd transmission event” (Table 1). Due to the close proximity of the final two farms where the animals resided (< 1 km), it was not possible to determine on which farm infection occurred. As mentioned previously, this was the only herd within the cluster of genetically near-identical isolates that had more than one isolate in the current WGS database, making it the only herd with enough data to assess a within-herd transmission event.

**Table 1:** The number of cases classified in each transmission pathway

Transmission Pathway	# Cases(%)	Homebred	bTB reactors
Within-herd transmission	2 (12.5)	0	0
Residual within-herd transmission	0 (0)	0	0
Local Area Transmission	7 (43.8)	4	2
Movement between herds	4 (25.0)	1	2
Movement within herds	3 (18.8)	0	0

### Residual within-herd transmission event

Only one herd fulfilling the inclusion criteria had WGS recorded from more than one breakdown, making it the only herd where a residual within-herd transmission event could be evaluated. The genetic divergence of the isolates excluded a residual within-herd transmission event in this herd.

### Local area transmission event

A total of seven (43.75%; 7/16) were classified as local area transmission events (Table 1; green points, Fig. 3, Plot B). These isolates were genetically near-identical (≤ 3 SNP) to other samples submitted from herds with land fragments located < 4 km from the lands registered to the submitting farms. The distance threshold was chosen based on epidemiological evidence indicating that badgers, a known wildlife reservoir for *M. bovis*, have an average ranging distance of < 2 km [33]. Furthermore, 4 km gave the best explanatory power in models measuring the relationship between bTB breakdown and bTB in neighbouring herds [23]. Therefore, a 4 km threshold was chosen to account for both direct transmission between neighbouring cattle herds and potential badger-mediated spread.

- Four of these seven animals were homebred (Green and black points, Fig. 3).
- Two animals were bTB reactors.

Transmission associated with cattle movement.

There were seven (43.75%; 7/16) isolates classified as transmission events associated with movement (Table 1), subdivided into:

#### Transmission event associated with cattle movement between herds (*n* = 4, 25%)

Four isolates were classified as transmission events associated with movement between herds. These isolates were obtained from animals that had moved between multiple herds before slaughter, with WGS data suggesting that transmission likely occurred prior to the final movement:

- Two cattle submitted from herds in Co. Wicklow and Co. Monaghan (pink points, Fig. 3), exhibited *M. bovis* isolates which were genetically distinct (> 50 SNPs) from the endemic sub-lineage local to their final herd of residence, indicating infection likely occurred outside their final herd.
- However, WGS data revealed that these animals carried isolates genetically near-identical (≤ 3 SNP) to those found local (< 4 km) to farms these animals had previously resided in. As the farm in Wicklow was > 200 km away, and the farm in Co. Monaghan was almost 20 km away, this strongly supports transmission of the pathogen occurred before their final move, i.e. a transmission event associated with between herd movement.
- One homebred animal submitted from a farm in Co. Donegal (pink points, Fig. 3), was classified as a transmission event associated with between herd movement despite no documented movement of that animal. This classification was based primarily on its spatial distance from other genetically closely related isolates from the sub-lineage’s likely core range in the Co. Armagh/Co. Monaghan region, suggesting an undetected epidemiological link. The latter could have arisen due to movement of an animal/or animals with undetected infection to the Co. Donegal herd location (or to herds local to it) from the Co. Armagh/Co. Monaghan core range and seeding infection in the new location.
- The fourth case (Black and white square, Fig. 3) involved a badger which was sampled > 15 km from farms with a genetically close (≤ 3 SNP) *M. bovis* isolate. There were no known epidemiological links between it and other cases in the study. However, given that badgers have an average ranging distance of < 2 km it is likely that this transmission event is associated with the movement of cattle between herds. Furthermore, as the isolates from the badgers and cattle were obtained in 2019 or later, it is not possible to deduce which direction the cattle travelled.

#### Transmission event associated with cattle movement within herds (*n* = 3, 18.75%)

Three isolates (Fig. 2, orange), were classified as “transmission events associated with movement within herds”:

- These isolates were collected from cattle from farms where the main farm fragment (home farm) was geographically distant (> 10 km, > 25 km & > 60 km) from other fragments (outside fragments) of land grazed by cattle in the submitting herd.
- The sampled cattle exhibited *M. bovis* isolates which were very genetically distinct (i.e. they are > 50 SNPs) from the endemic local sub-lineage surrounding (< 4 km) to the home farm, suggesting these infections did not originate in the main farm fragment.
- However, WGS data revealed that these animals carried isolates genetically nearly identical (≤ 3 SNP) to those found local (< 4 km) to the outside fragments of these farms; the transmission event may have occurred while animals grazed outside fragments.
- Movement between a herd’s land fragments are not required to be reported to AIM, but it may be possible to verify this with the herdowner. These undocumented movements may have played a role in this transmission event.

## Discussion

This study represents a framework for integrating WGS data with high-resolution epidemiological metadata to investigate recent *M. bovis* transmission dynamics in a bTB-endemic setting. Using a decision-tree framework applied to 16 near-identical isolates (≤ 3 SNP), this analysis demonstrates that it is possible to use the WGS of an *M. bovis* isolate to classify transmission events and attribute infection pathways in cattle herds when combined with classical epidemiological data. This work builds on the work of others, some of whom have used WGS to: reveal local transmission patterns of *M. bovis* in sympatric cattle and wildlife populations in England [26, 29]*,* Ireland [11] and elsewhere [28, 35] to identify *M. bovis* transmission clusters in Spain [36]; and to determine the source of *M. bovis* infection in cattle and livestock in New Zealand [10]. These publications, like our study, highlight the capacity of WGS data to enhance bTB surveillance, particularly in a country such as Ireland where high cattle density, large numbers of cattle movements, undisclosed infection, fragmented holdings, and wildlife reservoirs present persistent eradication challenges.

The classification of two of the sixteen isolates under study as being consistent with within-herd transmission events provides evidence for recent, direct or indirect spread between animals co-located in the same herd. The latter factor is a known problem in bTB eradication schemes in Britain and Ireland where imperfect diagnostic test sensitivities can prevent identification of all infected animals [7, 37], so that surveillance and control measures do not always curtail within-herd transmission events. The cases in this study, involving lesion-positive cattle submitted for slaughter at the same time, shared overlapping recent movement histories and genomic profiles, is consistent with recent infection events. The detection of such transmission events within a single breakdown episode illustrates how WGS can pinpoint transmission events within a herd, providing locally-tailored mitigation actions for veterinary field epidemiologists. When within-herd transmission events are identified, WGS provides a data-driven framework that identifies the need for herd-level control measures, such as movement restrictions, enhanced biosecurity, targeted diagnostic testing during prolonged outbreaks and risk-based trading. It is also important to investigate other potential transmission event pathways (namely, local transmission or movement associated transmission events) to investigate how infection entered the herd and to assess whether the appropriate control measures have been implemented, if the source is identified.

Given our pilot study’s small size, we cannot generalize our findings population-wide to infer how common intra-herd transmission events may be, or their contribution to the overall burden of disease in Ireland. However, as more data are collated, and our models are trained, specifically with more infected herds with > 1 isolate, the application of the framework described here would increasingly permit such inferences.

Finding “residual within-herd transmission events” below detectable limits in our dataset, based on a SNP divergence greater than the conservative threshold, suggests prior infection may have been cleared in the one herd with multiple WGS-positive breakdowns. This finding illustrates how integrating genomic and temporal data can provide evidence for a new infection, by (most probably) excluding “residual infection”, an important capability in herds with recurrent breakdowns. While limited by the small number of eligible herds, this result highlights the importance of temporal depth and multiple within-herd WGS isolates for understanding long-term persistent infection. The further evaluation of such herds is merited.

“Local area transmission events”, which are notoriously difficult to resolve, accounted for 43.75% of cases, emerging as the dominant event pathway in this study. The clustering of genetically near-identical *M. bovis* isolates in herds located within a 4 km radius provides strong evidence of spatially mediated transmission. However, the spatial extent of this cluster of near-identical isolates (approximately 20 km x 10 km), far exceeds the known average badger ranging distance of < 2 km [33]. While some badgers may range beyond this average distance, it is probable that whole-genome similarity would not be maintained if infection were transmitted sequentially across multiple social groups. This suggests that wildlife alone cannot fully explain the geographical extent of the near identical cluster of isolates. Instead, transmission may be sustained by a combination of direct cattle-to-cattle spread, shared environmental reservoirs, and wildlife vectors such as badgers [38, 39]. As temporal depth of the current WGS dataset is shallow there is a risk that, due to fragmentation in Irish farms, local transmission events have occurred because of within-herd movement of infected cattle. As the WGS dataset acquires more temporal depth from sequencing of isolates across a wider time frame it may be easier to understand and resolve this complexity.

Our findings suggest the movement of undetected but infected cattle plays a role in fuelling short-range bTB transmission. Several homebred animals within these clusters carried *M. bovis* isolates genetically near-identical to those found in neighbouring herds. Given the lack of recorded movement for these cattle, this strongly supports exposure, potentially via shared boundaries, contaminated grazing land, or wildlife traversing farm borders. The presence of such animals points to a complex network of largely unobserved transmission routes that operate below the resolution of standard epidemiological “test and trace” actions.

Homebred animals, in this context, serve as critical spatial anchors within local transmission event networks. Because they remain within a single herd from birth to slaughter, their infection can be confidently linked to specific geographic locations and local epidemiological conditions. Indeed, the location of badger captures, due to their tendency to reside in small, localised areas [33, 38] potentially also serve as spatial anchors. In this study, the spatial distribution of homebred animals carrying genetically identical isolates helped delineate a defined “home range” or kernel for the transmission cluster; a geographic footprint of ongoing infection. These spatially stable clusters, centred around homebred animals, stand in contrast to the broader dispersal patterns observed in cattle with more complex residency histories. While mobile animals blur transmission boundaries due to multiple potential exposure points, homebred cases allow for more precise spatial attribution, making them valuable for mapping infection persistence.

The integration of temporal depth into WGS datasets would further enhance analysis of home ranges, enabling dynamic monitoring of home ranges over time. By analysing isolate clustering across multiple years, researchers could determine whether specific clusters are expanding, contracting, fragmenting, or shifting across the landscape [40]. These trends would offer vital insights into the impact of local interventions, the emergence of new hotspots, or the silent reintroduction of infection via the movement (recorded or unrecorded) of cattle with undetected infection, or wildlife activity. Furthermore, linking this spatio-temporal genomic data with land-use patterns, movement intensity, and environmental variables could illuminate key drivers of range dynamics, such as increased cattle turnover, fragmented land management, or habitat changes affecting wildlife behaviour.

Homebred animals are not merely endpoints in surveillance data, they are sentinels of localised transmission pressure. When embedded within a genomic epidemiology framework, they offer the geographic resolution and interpretive clarity needed to identify persistent infection zones, characterise local transmission dynamics, and evaluate the effectiveness of area-based bTB control strategies with forensic precision.

Cattle movement-associated transmission events were equally prominent, accounting for 43.75% of classified cases. Genomic data supported the hypothesis that infection was acquired prior to arrival in its final herd, as WGS data from isolates obtained from the final herd, or other herds local to it (< 4 km), exhibited insufficient genomic similarity to the isolate from the animal being investigated for an epidemiological link to be considered. This illustrates the limitations of traditional contact tracing, where infection may be attributed to the wrong source based on residency alone. Moreover, the inclusion of within-herd movement between fragmented land parcels, not reported in AIM, as a significant source of infection (18.75% cases) reflects an important blind spot in existing surveillance infrastructure. These undocumented movements present a biosecurity vulnerability in the national eradication strategy, particularly in regions with high land fragmentation and shared grazing arrangements.

A particularly salient finding of this study is the detection of WGS-confirmed transmission events involving animals that tested negative in bTB surveillance tests, exposing a limitation of current diagnostics in detecting bTB infection. These undetected carriers pose a substantial risk to eradication efforts, as they may act as silent disseminators of infection across herds and regions. Further evidence of this diagnostic blind spot is illustrated by the detection of a single homebred animal in Co. Donegal carrying an *M. bovis* isolate genetically near-identical to others in a very spatially distant (> 100 km) cluster. Assuming the recorded movement history for this homebred animal is complete and accurate, this animal acquired infection in Co. Donegal, implying that undetected infection was introduced to Co. Donegal from the cluster’s home range via movement of cattle with undetected infection. This case exemplifies the forensic utility of WGS as a retrospective investigative tool. By revealing hidden epidemiological links that conventional tracing methods would likely miss, WGS offers a powerful means of flagging potential long-distance transmission events and identifying areas, or herds, that may warrant further investigation. These findings also highlight the benefit of testing history traceability and biosecurity risks associated with cattle movements between herds.

### Limitations

Despite the clear power of WGS in elucidating transmission dynamics, certain limitations of this study must be acknowledged. The small sample size (*n* = 16) of this pilot study limits the generalisability of the findings and undoubtedly results in a sampling bias as only one farm was selected as the focus of the outbreak investigation. This is an inherent limitation of a study with a small sample size, such as a pilot study. This could be addressed through larger, more extensive studies conducted across broader spatial and temporal scales. Larger studies would most likely highlight how bTB epidemiology varies in time and space across the landscape. This would undoubtedly lead to refinements of the decision tree proposed here, potentially location specific distance cut offs. The latter being dependent on establishment of island wide, robust, home ranges for specific *M. bovis* sub-lineages and clades, which will require much denser sampling.

Another challenge lies in classifying transmission events that satisfy the criteria for multiple pathways. In situations involving within-herd transmission events or residual transmission events, it is important to investigate how infection entered the herd under study i.e. via local area transmission and/or transmission associated with cattle movements. To address this, we ranked the transmission event pathways according to the level of control measures required. In this pilot study, evidence of within-herd transmission was identified in the index herd, warranting herd-level targeted controls. There was also evidence of local transmission events involving herds surrounding to the final two holdings in which these animals resided. The index animals are not assigned a local area transmission event classification, as it is likely that both the index herd and their previous herd would be included in control strategies triggered by neighbouring herds’ local transmission event classification. However it is important for field epidemiologist to ensure that the appropriate control strategies are implemented. It is also be noted that some aspects of the decision tree may always require human intervention to forensically investigate discrepancies in evidence between WGS and epidemiological data [29].

The reliance on *M. bovis* culture-positive samples restricts WGS data to a subset of the infected population, typically those with visible lesions detected at slaughter. This inherently biases the dataset toward more advanced infections and introduces a temporal disconnect between infection and detection. As a result, WGS offers a retrospective, incomplete view of transmission event chains and cannot, on its own, capture the real-time spread of infection within or across herds.

Additionally, the accuracy of life events metadata, essential for interpretating genomic patterns, is constrained by gaps in records, potential misclassification and lack of granularity in within-herd movements and the role of farm fragmentation. These challenges highlight the need for continuous improvement in data capture systems and a cautious interpretation of WGS findings in isolation. Another inherent challenge is the slow and variable mutation rate of *M. bovis*, a slow growing pathogen [5, 12]. This results in homogenous genetic isolates persisting over variable timescales, making it difficult to infer recent transmission events based solely on SNP distance. However, this limitation can be mitigated, though not eliminated, by integrating WGS with high-resolution life events data. In this study, such integration proved essential in assigning plausible transmission event pathways despite genomic homogeneity.

Another obvious limitation arises from the geographical context of this cluster, whose inferred home range spans the border between the Republic of Ireland and Northern Ireland. The limited availability of detailed metadata from Northern Ireland hampers full resolution of cross-border transmission event pathways. This highlights the value of increased cross-border collaboration across the island of Ireland to understand and manage transborder bTB transmission more effectively.

Nonetheless, the integration of WGS into routine regional veterinary epidemiology represents a significant advancement for bTB surveillance in Ireland. The decision-tree framework developed in this study offers a scalable, standardised, and transparent methodology for classifying transmission event pathways at the outbreak level. However, the heterogeneity of farm practices in Ireland means interpretation at the outbreak level should always incorporate the nuances associated with the herds involved.

### Further research

Beyond its utility in retrospective outbreak reconstruction, the identification and classification of transmission event pathways may have several broader applications with direct relevance to Ireland’s bTB eradication programme. There is potential for a fully or partly automated transmission event pathway classification approach which would facilitate further validation of the approaches outlined here via a much larger dataset, possibly in a longitudinal study. An interactive rule-based classification, based on the decision tree developed in this study should be straightforward to implement, as it relies on predefined thresholds and logical conditions that can be coded into the existing framework without requiring extensive computational resources. This could take the form of an interactive decision-support system where users input case details, and the tool could provide ranked transmission scenarios based on the forensic framework. This could also involve a dynamic interface where users adjust parameters (e.g., SNP distance threshold) and observe changes in classification and perhaps automated report generation summarising the likely transmission pathway and supporting evidence.

That being said, the decision tree developed here requires further testing in a larger study population prior to automation, during which necessary refinements could be made. Nevertheless, developing an automated decision tool could be useful, as it could be applied to a preselected number of herds to ascertain the relative contribution of different transmission event pathways across a broader geographical area. If such a tool were validated, it could be applied to the entire Irish WGS database and may be able to provide relevant parameters for infectious disease modelling. If it were applied at a regional level, it may enable regional monitoring of spatial movement in home ranges and emerging transmission event patterns, providing evidence to support efficient allocation of resources. If the automated decision tool was applied to a smaller region (Chronic bTB blackspots) it may provide evidence for tailored farm-specific intervention strategies and efficient allocation of resources locally. Such a tool would also have the potential to monitor areas surrounding high risk herds (e.g., CFUs) to detect if *M. bovis* strains introduced via long range movements have spilled over into local herds.

## Conclusion

WGS has the potential to enhance Ireland’s bTB eradication programme by attributing infection sources, identifying transmission event pathways and quantifying transmission rates [13]. The decision tree developed in this pilot study provides evidence that WGS, when integrated with spatial, lifespan and movement metadata, offers a means of characterising *M. bovis* transmission event pathways. The findings confirm that within-herd transmission, local area transmission, and both documented and undocumented cattle movements all play a role in sustaining bTB in Ireland. Automating this methodology and applying it to a larger dataset in a longitudinal study would allow further understanding of the risks associated with each event pathway. Importantly, the role of undetected carriers, i.e. animals testing negative at SICTT yet implicated in transmission events, highlights the critical diagnostic gap in current surveillance approaches and thus the importance of enhanced methods of bioexclusion, such as risk-based trading.

Although bTB incidence remains high in both the Republic of Ireland and Northern Ireland, integrating standardised methodologies to temporally deep WGS is already warranted to support eradication efforts. When bTB incidence declines and the drive towards eradication efforts intensifies, tools to integrate WGS data and epidemiological metadata may be even more valuable. This is because WGS technology has the potential to inform, evaluate, and enhance disease modelling, inform targeted interventions, and ultimately optimise the allocation of resources within the national eradication programme. Although a cost–benefit analysis is beyond the scope of this study, future work should assess whether efficiencies gained through more targeted interventions and better allocation of resources outweigh the cost of routine WGS implementation. Importantly, the benefit of WGS, especially its temporal and spatial depth, will be greatest as incidence of bTB declines and endemic regions become geographically discrete.

Even though harnessing the utility of WGS data in bTB transmission dynamics is in its infancy, there is much potential for automation. The decision-tree framework developed here could be a foundation for future analyses, ensuring consistent and scalable classification which will provide information on transmission events across expanding WGS datasets. When paired with improved data capture systems and integrated surveillance, this approach will strengthen Ireland’s capacity to respond to persistent infection hotspots, mitigate reinfection risk, and accelerate progress towards bTB eradication.

WGS will therefore enhance the efficacy of the bTB Eradication Programme in Ireland and can be harnessed to attribute infection sources and identify transmission pathways, as well as to quantify transmission rates across species.

## Supplementary Information

Supplementary Material 1.

Supplementary Material 2.

## Data Availability

The datasets accessed during this study are available from the Department of Agriculture, Food and the Marine, but are subject to data protection regulations (https://datacatalogue.gov.ie/organization/department-of-agriculture-food-and-the-marine).
