# Supplementary material for: Integrating whole-genome sequencing and epidemiology to characterise Mycobacterium bovis transmission in Ireland: a proof of concept
Source: Ir Vet J. 2025 Dec 1;79:3. doi: 10.1186/s13620-025-00321-3 (PMC12771759; doi:10.1186/s13620-025-00321-3)

Figure 1: Homebred Pathway.

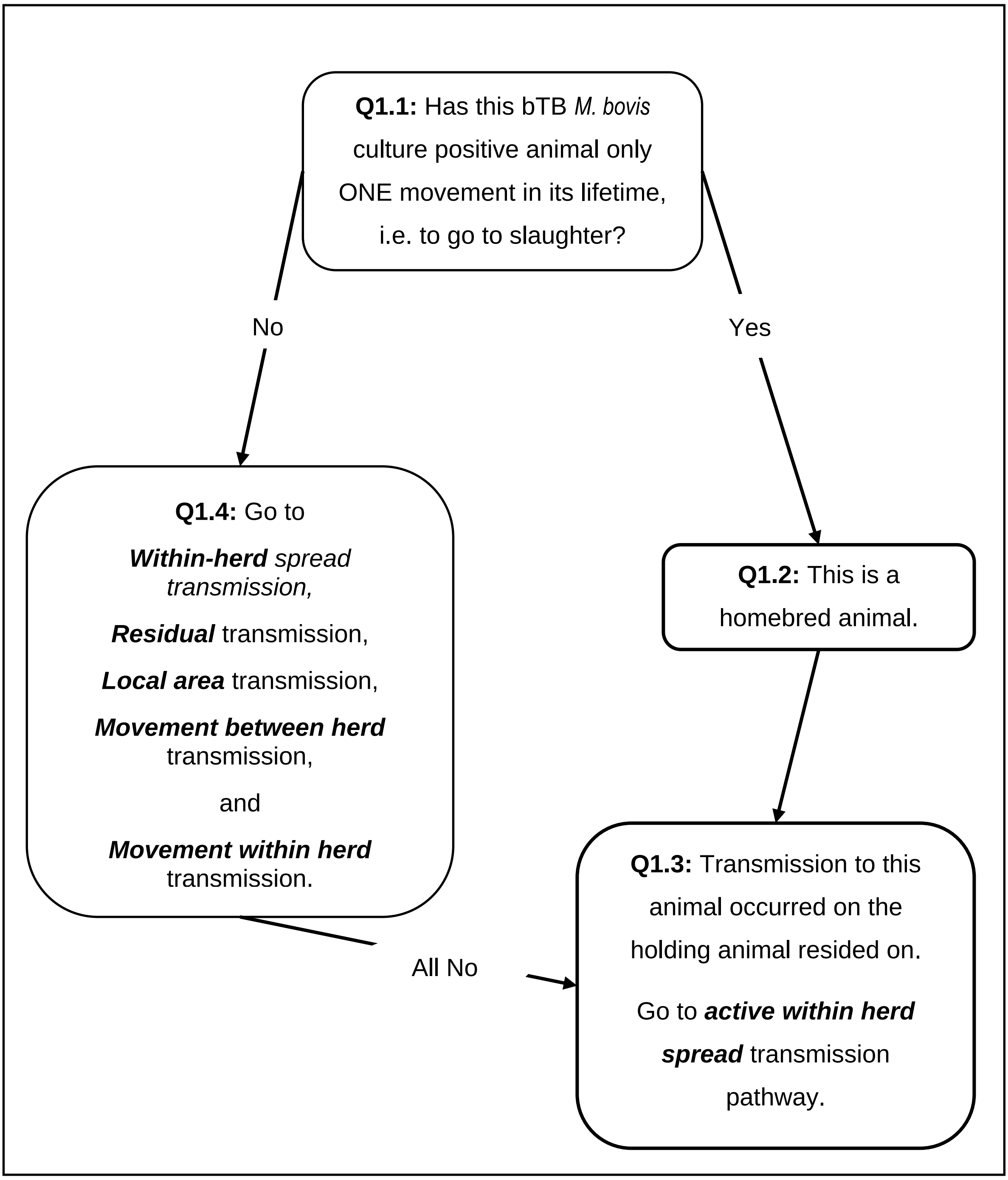

Figure 2: Within Herd spread Transmission pathway in a highly endemic population.

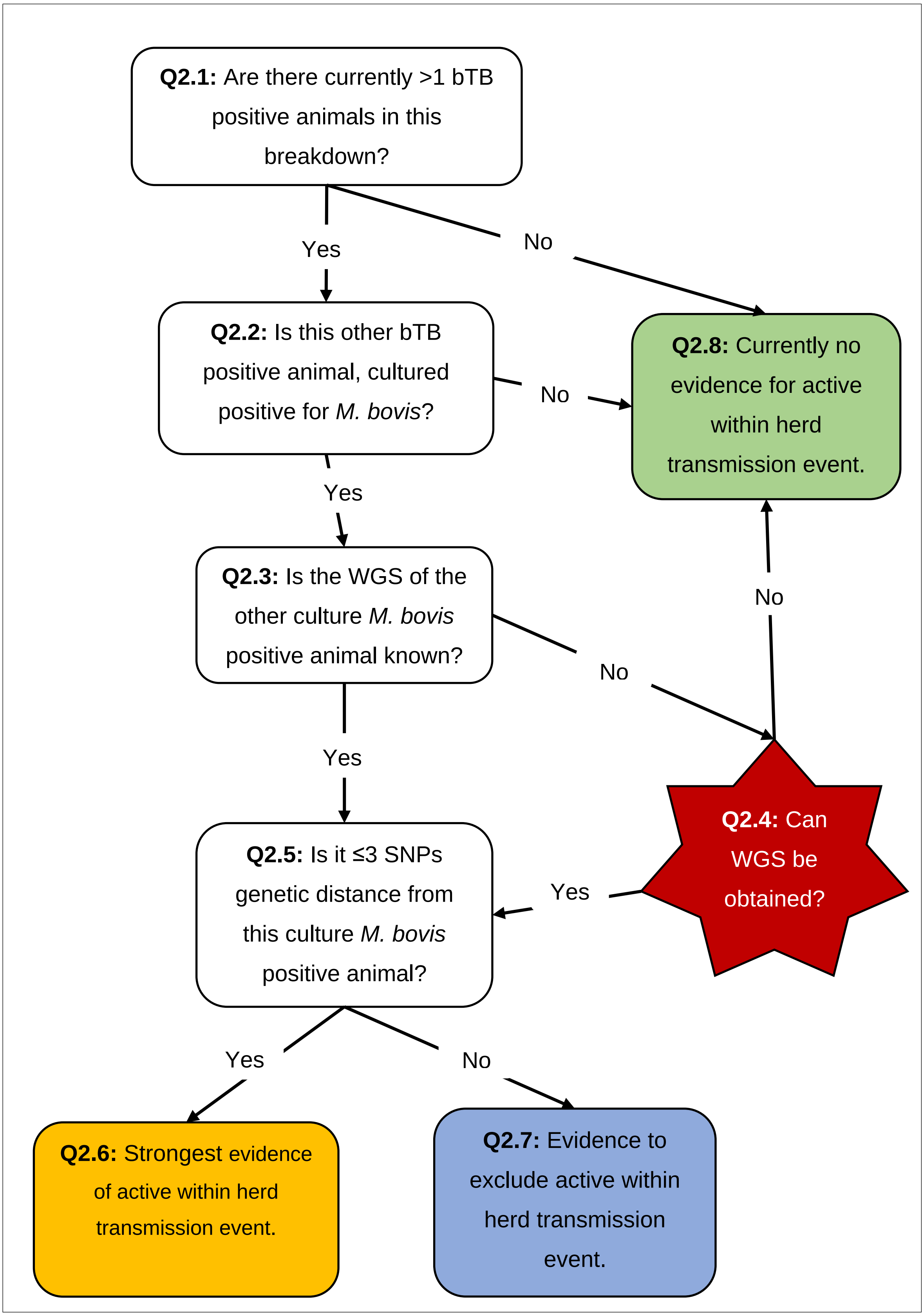

Figure 3: Residual Within Herd Transmission Pathway in a highly endemic population…

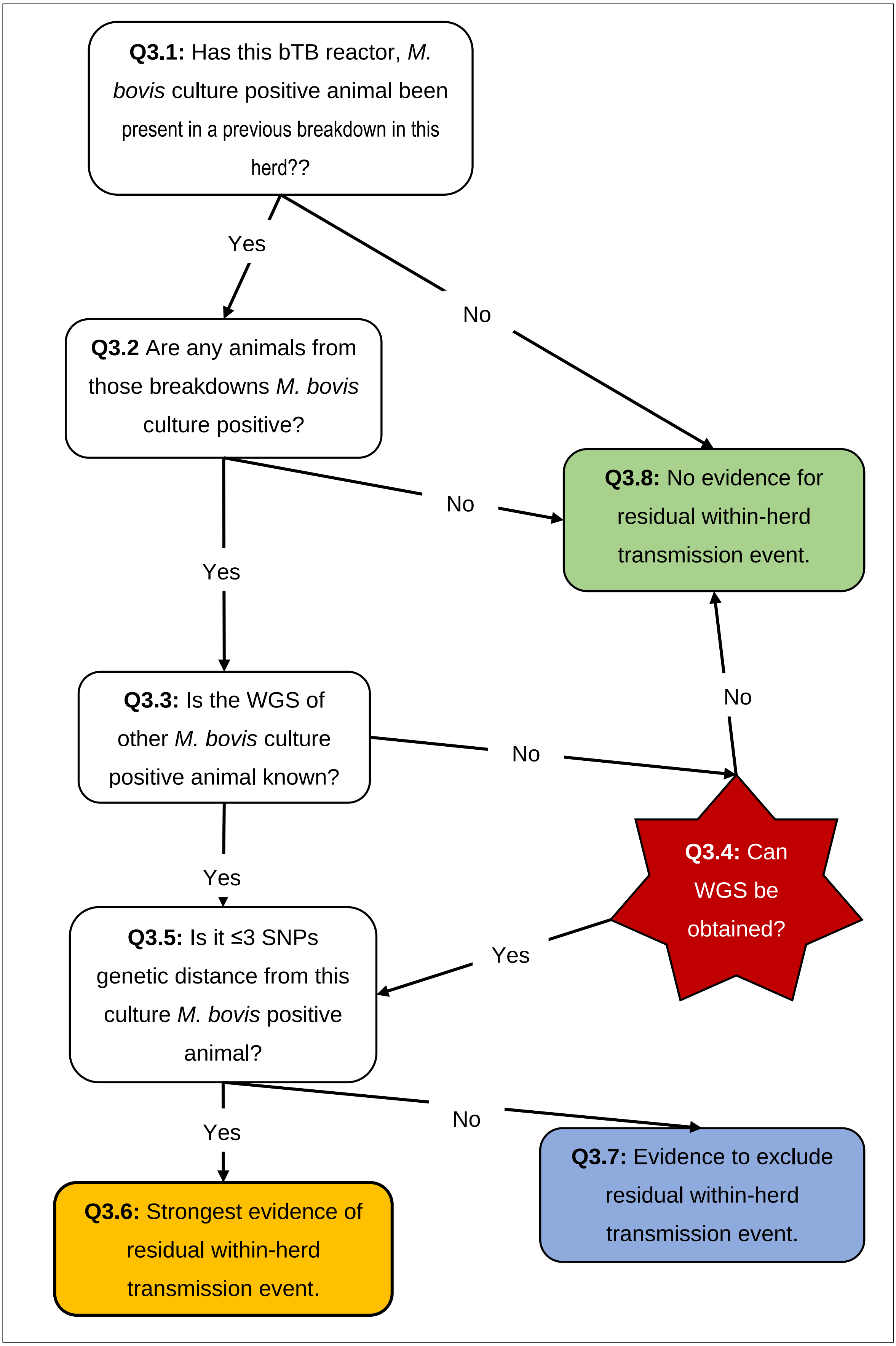

Figure 4: Local area Transmission Pathway in a highly endemic population.

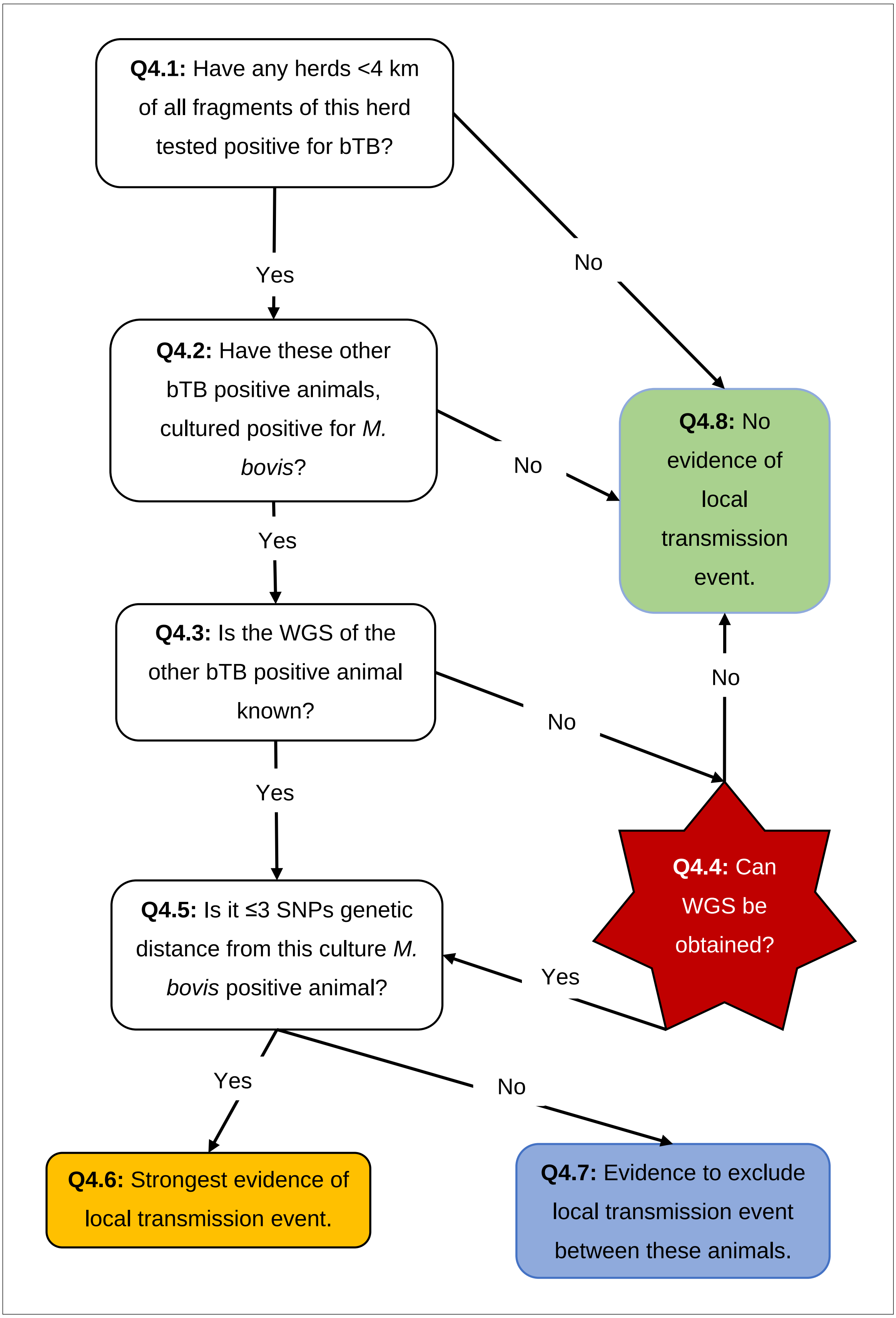

Figure 5: Movement between herd transmission pathway in a highly endemic population.

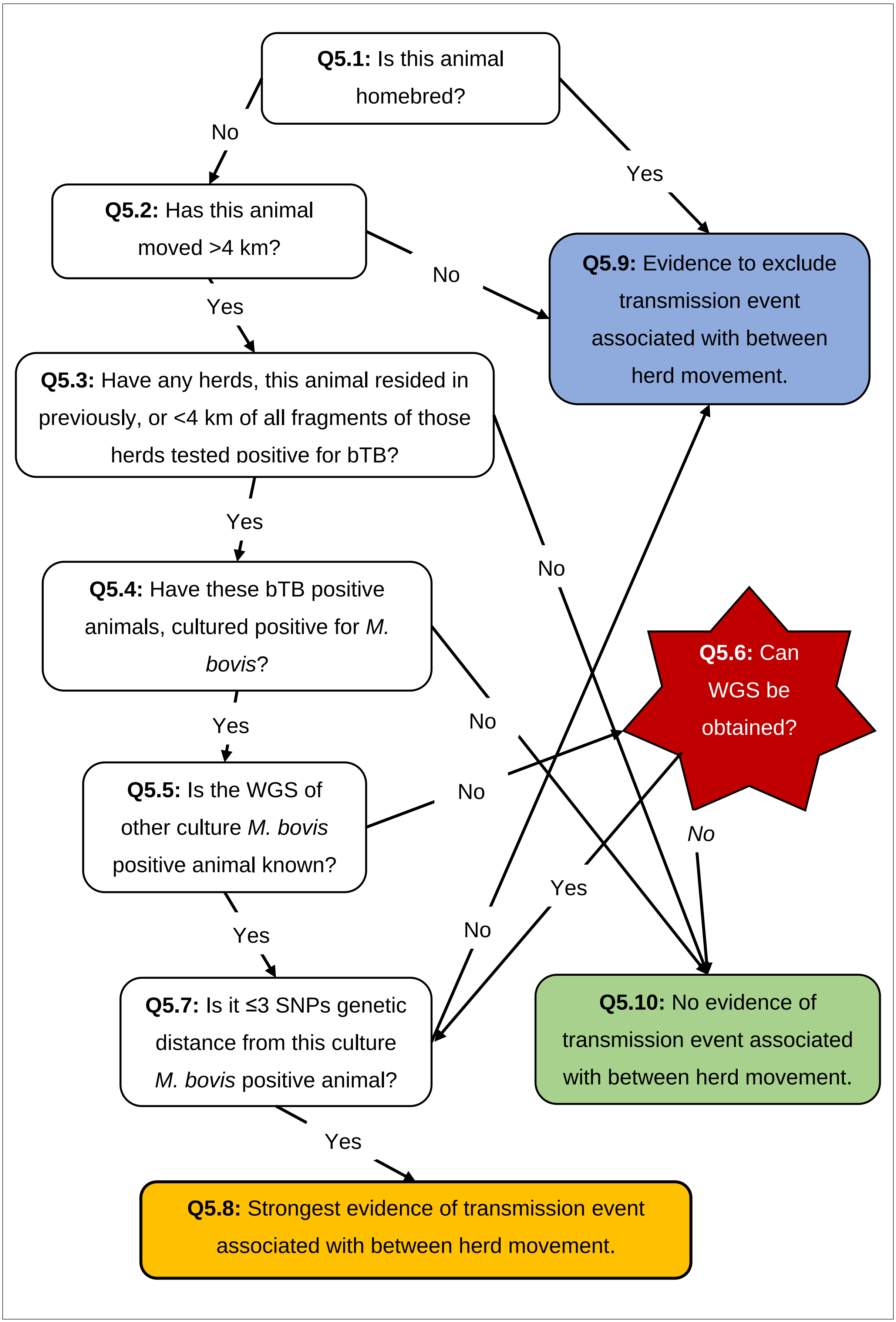

Figure 6: Movement within herd transmission pathway in a highly endemic population.

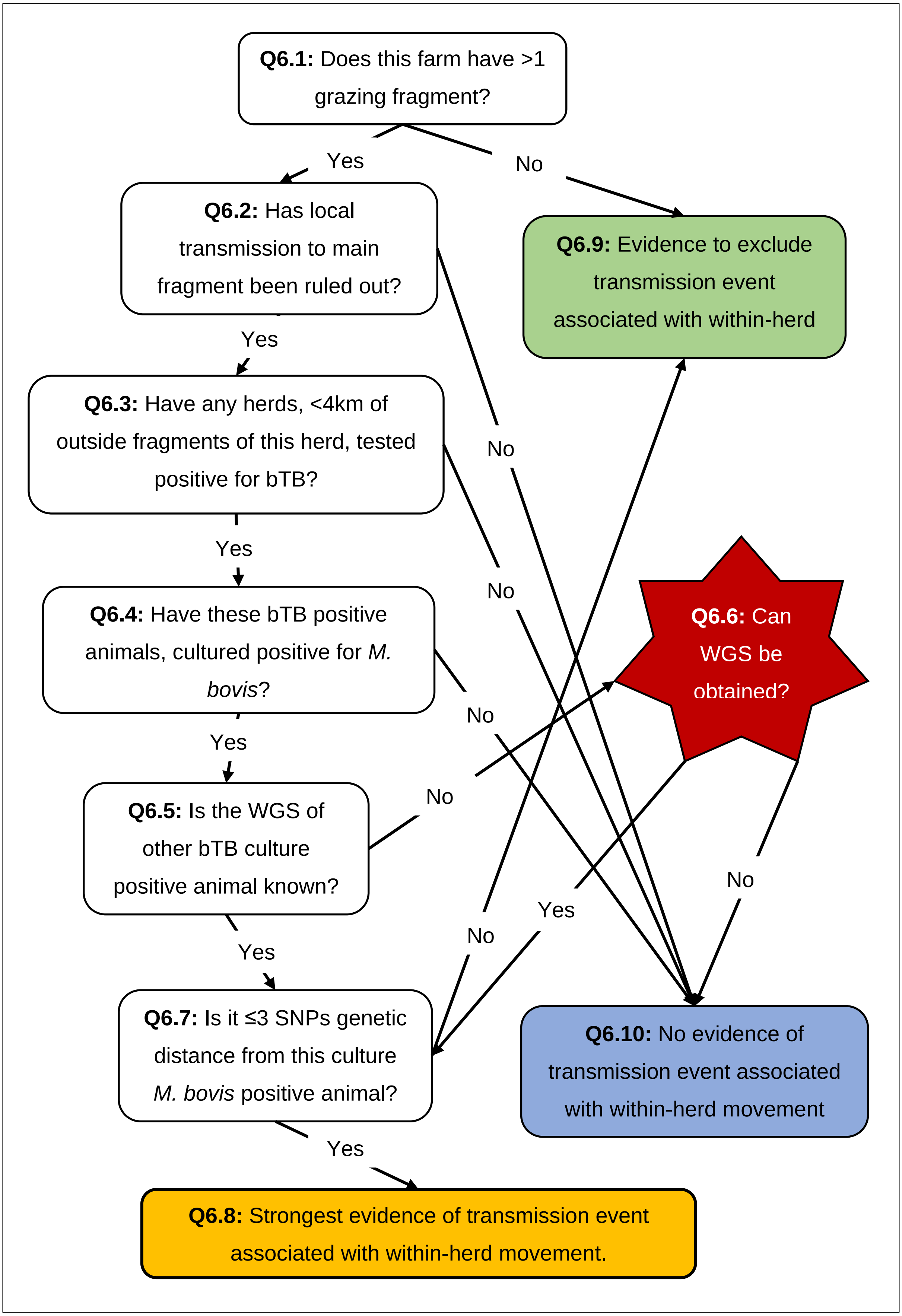

Supplement: Supplementary file 1 — Supplementary Material 1. [file 13620_2025_321_MOESM1_ESM.pdf]
