# Supplementary material for: Integrating whole-genome sequencing and epidemiology to characterise Mycobacterium bovis transmission in Ireland: a proof of concept
Source: Ir Vet J. 2025 Dec 1;79:3. doi: 10.1186/s13620-025-00321-3 (PMC12771759; doi:10.1186/s13620-025-00321-3)
Supplement: Supplementary file 2 — Supplementary Material 2. [file 13620_2025_321_MOESM2_ESM.docx]

| **Key** | **Question** | **Yes** | **NO** |
| --- | --- | --- | --- |
| **Homebred Pathway** | | | |
| 1.1 | Has this *M. bovis* culture positive animal exactlyONE movement in its lifetime, i.e. to go to slaughter? | Go to 1.2 | Go to 1.4  *So 1.2=0, 1.3 =0 5.1=0* |
| 1.2 | This is a homebred animal. | Go to 1.3 so *1.3=1* |  |
| 1.3 | Transmission to this animal occurred on the holding this animal resided on. |  |  |
| 1.4 | Go to Within -herd spread, residual, local area, movement between herds and movement within herd transmission pathways. |  |  |
| **Within-Herd transmission event in a highly endemic population.** | | | |
| 2.1 | Are there currently >1 bTB positive animals in this breakdown? | Go to 2.2 | Go to 2.8 |
| 2.2 | Is this other bTB animal, *M. bovis* culture positive? | Go to 2.3 | Go to 2.8 |
| 2.3 | Is the WGS of the other *M. bovis* culture positive animal known? | Go to 2.5 | Go to 2.4 |
| 2.4 | Can WGS be obtained? | Go to 2.5 | Go to 2.8 |
| 2.5 | Is it ≤ 3SNPs genetic distance from this *M. bovis* culture positive animal? | Go to 2.6 | Go to 2.7 |
| 2.6 | Strongest evidence of active within herd transmission event. |  |  |
| 2.7 | Evidence to exclude active within herd transmission event. |  |  |
| 2.8 | Currently no evidence of active herd transmission event. |  |  |
| **Residual within herd transmission event in a highly endemic population.** | | | |
| 3.1 | Has this bTB reactor, *M. bovis* culture positive animal been present in a previous breakdown in this herd? | Go to 3.3 | Go to 3.2 |
| 3.2 | Are any animals from those breakdowns Culture positive for *M. bovis*? | Go to 3.3 | Go to 3.8 |
| 3.3 | Is the WGS of the other *M. bovis* culture positive animal known? | Go to 3.5 | Go to 3.4 |
| 3.4 | Can WGS be obtained? | Go to 3.5 | Go to 3.8 |
| 3.5 | Is it ≤ 3SNPs genetic distance from this *M. bovis* culture positive animal? | Go to 3.6 | Go to 3.7 |
| 3.6 | Strongest evidence of residual within-herd transmission event. |  |  |
| 3.7 | Evidence to exclude residual within-herd transmission event. |  |  |
| 3.8 | No evidence of residual within-herd transmission event. |  |  |
| **Local area transmission pathway in a highly endemic population.** | | | |
| 4.1 | Have any herds <4 km of all fragments of this herd tested positive for bTB? | Go to 4.2 | Go to 4.8 |
| 4.2 | Have these other bTB positive animals, cultured positive for *M. bovis* ? | Go to 4.3 | Go to 4.8 |
| 4.3 | Is the WGS of the other bTB culture positive animal known? | Go to 4.5 | Go to 4.4 |
| 4.4 | Can WGS be obtained? | Go to 4.5 | Go to 4.8 |
| 4.5 | Is it ≤ 3SNPs genetic distance from this *M. bovis* culture positive animal? | Go to 4.6 | Go to 4.7 |
| 4.6 | Strongest evidence of local transmission event. |  |  |
| 4.7 | Evidence to exclude local transmission events between these animals. |  |  |
| 4.8 | No evidence of local transmission event. |  |  |
| **Movement between herd transmission pathway in a highly endemic population.** | | | |
| 5.1 | Is this animal homebred? | Go to 5.9 | Go to 5.2 |
| 5.2 | Has this animal moved >4 km? | Go to 5.3 | Go to 5.9 |
| 5.3 | Have any herds, this animal resided in previously, or <4 km of all fragments of those herds, tested positive for bTB? | Go to 5.4 | Go to 5.10 |
| 5.4 | Have these bTB positive animals cultured positive for *M. bovis*? | Go to 5.5 | Go to 5.10 |
| 5.5 | Is the WGS of the other bTB culture positive animal known? | Go to 5.7 | Go to 5.6 |
| 5.6 | Can WGS be obtained? | Go to 5.7 | Go to 5.10 |
| 5.7 | Is it ≤ 3SNPs genetic distance from this *M. bovis* culture positive animal? | Go to 5.8 | Go to 5.9 |
| 5.8 | Strongest evidence of transmission event associated with between herd movement. |  |  |
| 5.9 | Evidence to exclude transmission event associated with between herd movement. |  |  |
| 5.10 | No evidence of transmission event associated with between herd movement. |  |  |
| **Movement within herd transmission pathway in a highly endemic population.** | | | |
| 6.1 | Does this farm have >1 grazing fragment? | Go to 6.2 | Go to 6.9 |
| 6.2 | Has local transmission to main fragment been ruled out? | Go to 6.3 | Go to 6.10 |
| 6.3 | Have any herds, <4km of outside fragments of this herds, tested positive for *M. bovis*? | Go to 6.4 | Go to 6.10 |
| 6.4 | Have these bTB positive animals cultured positive for bTB? | Go to 6.5 | Go to 6.10 |
| 6.5 | Is the WGS of the other bTB culture positive animal known? | Go to 6.7 | Go to 6.6 |
| 6.6 | Can WGS be obtained? | Go to 6.7 | Go to 6.10 |
| 6.7 | Is it ≤ 3SNPs genetic distance from this *M. bovis* culture positive animal? | Go to 6.8 | Go to 6.9 |
| 6.8 | Strongest evidence of transmission event associated with within-herd movement. |  |  |
| 6.9 | Evidence to exclude transmission event associated with within-herd movement. |  |  |
| 6.10 | No evidence of transmission event associated with within-herd movement. |  |  |
